# Clinical Factors to Predict Difficult Ureter during Ureteroscopic Lithotripsy

**DOI:** 10.1155/2023/2584499

**Published:** 2023-02-02

**Authors:** Masashi Imano, Tadashi Tabei, Hiroki Ito, Junichi Ota, Kazuki Kobayashi

**Affiliations:** ^1^Department of Urology, Yokosuka Kyosai Hospital, 1-6 Yonegaghama-Dori, Yokosuka, Kanagawa, Japan; ^2^Department of Urology, National Hospital Organization Yokohama Medical Center, 3-60 Harajuku, Totsuka, Yokohama, Kanagawa, Japan; ^3^Department of Urology, National Defense Medical College, 3-2 Namiki, Tokorozawa, Saitama, Japan; ^4^Department of Urology, Yokohama City University Hospital, 3-9 Fukuura, Kanazawa, Yokohama, Kanagawa, Japan; ^5^Department of Urology, Yokohama Municipal Citizen's Hospital, 1-1 Mitsuzawa-Nishicho, Kanagawa, Yokohama, Kanagawa, Japan

## Abstract

**Objective:**

To identify risk factors for difficult ureters during ureteroscopic lithotripsy and to determine the appropriate indications for preoperative stenting.

**Methods:**

We retrospectively analyzed 156 ureteroscopic procedures for upper urinary tract stones after excluding those with preoperative stenting or percutaneous nephrostomy. Traceability of the ureter was assessed by two urologists. Traceability was defined as positive if either or both urologists discerned the ureter in all slices on preoperative plain computed tomography. Patients' backgrounds were compared between the nondifficult ureter and difficult ureter groups. A multivariate logistic regression model was used to evaluate the relationships between difficult ureters and other clinical factors.

**Results:**

Of 156 patients, 31 (19.9%) were classified into the difficult ureter group. The positive traceability was higher in the nondifficult ureter group (48.3% vs. 83.2%, *P* < 0.001). The major axis was smaller in the difficult ureter group than in the nondifficult ureter group (8.8 ± 3.9 mm vs. 10.9 ± 4.5 mm, *P* < 0.018). A major axis <8 mm (odds ratio: 4.495, 95% confidence interval: 1.791–11.278, and *P*=0.001), negative traceability (odds ratio: 7.565, 95% confidence interval: 2.693–21.248, and *P* < 0.001), smoking status (odds ratio: 3.196, 95% confidence interval: 1.164–8.773, and *P*=0.024), and absence of diabetes mellitus (odds ratio: 5.813, 95% confidence interval: 1.121–30.142, and *P*=0.036) were identified as independent predictors of difficult ureters on multivariate logistic regression analysis.

**Conclusion:**

Patients with smaller stones, negative traceability, ongoing tobacco consumption, and absence of diabetes mellitus were at higher risk of difficult ureters. In these patients, preoperative stenting may be considered.

## 1. Introduction

Improvements in endoscope technology and peripheral devices for ureteroscopy (URS) for upper urinary tract stones enabled procedures more effective than shockwave lithotripsy (SWL) [1]. In particular, the development of smaller endoscopes and ureteral access sheaths had large impacts on facilitating this surgical procedure [2, 3]. The European Association of Urology and the American Urological Association have recommended URS as a first option for stones smaller than 10 mm of upper urinary tract lithiasis [1, 4, 5]. However, urologists sometimes encounter narrow and difficult ureters (DUs) while attempting to ascend the access sheath or endoscope in some patients [6]. Preoperative stenting is meant to solve this difficult condition [7, 8] with several favorable effects on URS, such as shortening the procedure time [9], improving the stone-free rate [10], and reducing the risk of injury to the ureter [11]. On the other hand, indwelling ureteral stents are associated with significant morbidity and poor quality of life in 80% of patients [12], and preoperative stenting should only be indicated for appropriately selected patients [1]. The identification of candidates for preoperative stenting before URS remains obscure. This study aimed to investigate predictive factors for DU for appropriate patient selection for preoperative stenting.

## 2. Materials and Methods

The Institutional Review Board of the Yokosuka Kyosai Hospital approved this study (approval number: 20–35). The requirement for written informed consent was waived by the institutional review board because all data were anonymized and collected retrospectively. Patients' consent was obtained upon an opt-out basis as communicated through notice boards across Yokosuka Kyosai Hospital. All study procedures were carried out following the Declaration of Helsinki principle.

### 2.1. Patient Selection

For urinary stones <20 mm in diameter, SWL and URS are usually proposed. For renal stones >20 mm in diameter, PCNL is offered as the first option, with URS offered as second-line therapy. The final decisions were borne of discussion between urologists and patients. In this study, we excluded patients with prior preoperative stenting or percutaneous nephrostomy. Between April 2017 and January 2020, 156 URS procedures for upper urinary tract stones were analyzed.

### 2.2. Surgical Techniques

Our surgical procedures have been previously described [13, 14]. In summary, intravenous antibiotic prophylaxis based on the urine culture was administered upon induction of general anesthesia until postoperative day 1. In the lithotomy position, we initially performed cystoscopy to rule out intravesical lesions. A 6.4/7.8 Fr semirigid ureteroscope (WA29040A, Olympus, Japan) was inserted up to the most distal stone or the ureteropelvic junction. Ureteral access sheaths (Bi-flex Evo, 10/12 or 12/14 Fr, Rocamed) were placed through a guidewire in cases of renal stones. A 270 *μ*m Holmium: Yttrium–Aluminum–Garnet laser (Dornier Medias H UroPulse; Dornie) was used to fragment stones through a semirigid or flexible ureteroscope (Olympus P-6, Olympus). 1.5 Fr tipless nitinol baskets (Dormia, Coloplast) were used to extract stone fragments. A ureteral stent was placed after those procedures upon the surgeon's discretion. A 14 Fr urethral catheter was placed in all cases.

### 2.3. Definition of DU

A DU was defined as narrowing encountered upon ascending the ureteroscope or ureteral access sheath, complicating the conduct of a complete URS procedure (from fragmentation to extraction), without any prior history of urinary obstruction and signs and symptoms of back pain or hydronephrosis before urolithiasis was diagnosed. If DU was diagnosed upon URS and fragmentation was not possible, stenting was performed instead of ureteral dilatation and a second procedure was scheduled 2-3 weeks later. If DU was diagnosed upon URS and fragmentation was possible, a second procedure or stent removal was considered according to the amount of residual stone.

### 2.4. Preoperative and Postoperative Imagings

Plain computed tomography (CT) was obtained at every 5 mm axial slice and was managed by Century Enterprise Web V3.0 (GE Healthcare) for all patients before surgery. The major axis was defined as the longest diameter of all stones. “Ureteral traceability” was assessed by two urologists (TT and IH) with over 10 years of urological experience. They were blinded to the patient's information and the treatment outcomes. Ureteral traceability was defined as “positive” when either or both urologists were able to discern the ureter of the treated side at every CT slice from the pelvic-ureter junction to the vesicoureteric junction. Stone-free status was defined as the absence of residual fragments upon low-dose CT taken 2-3 weeks after stent removal.

### 2.5. Statistical Analyses

Clinical and stone-specific variables between the non-DU and DU groups were compared. These variables included sex, age, body mass index, laterality of the stone, stone location, major axis, maximum Hounsfield unit, hydronephrosis, ureteral traceability, history of ipsilateral urolithiasis on the affected side, abdominal surgery, diabetes mellitus (DM), smoking habit, hypertension (HT), dyslipidemia (DL), and cardiovascular or cerebrovascular events.

Continuous variables were described as means (standard deviations) and were compared using Student's *t*-test. Categorical variables were expressed as numbers (percentages) and were compared using chi-squared analysis. The cutoff value of the major axis for DU presence was determined with a receiver operating characteristic (ROC) curve analysis. A multivariate logistic regression model was used to identify risk factors predictive of DU in our patients. The level of significance was set at a *P* value <0.05, and all analyses were performed using SPSS (version 19, Chicago, IL, United States).

## 3. Results

### 3.1. Comparison between DU and Non-DU Groups

Of the 156 cases of URS included in our analysis, 125 were classified as non-DU and 31 (19.9%) were classified as DU. Fragmentation alone could be achieved in 17 of the 31 patients, while 14 completed the procedure with an indwelling ureteral stent.

The clinical and stone-specific variables are summarized in Table 1 for the DU and non-DU groups. The variables were comparable between the two groups, except for the following: positive ureteral traceability was higher in the non-DU group (48.3% (DU) vs. 83.2% (non-DU), *P* < 0.001). The major axis was smaller in the DU group than in the non-DU group (8.8 ± 3.9 mm vs. 10.9 ± 4.5 mm, *P* < 0.018).

### 3.2. Multivariate Analysis

The ROC curves identified the cutoff value of the major axis associated with DU presence as 8 mm. Multivariate logistic regression analysis identified these as independent factors of DU presence, namely, a major axis <8 mm (*P*=0.001, odds ratio (OR): 4.495, and 95% confidence interval (CI): 1.791–11.278), negative traceability (*P* < 0.001, OR: 7.565, and 95% CI: 2.693–21.248), smoking status (*P*=0.024, OR: 3.196, and 95% CI: 1.164–8.773), and absence of DM (*P*=0.036, OR: 5.813, and 95% CI: 1.121–30.142) (Table 2).

### 3.3. Outcome of URS

Of the 14 patients for whom even fragmentation could not be performed, 12 underwent URS 2-3 weeks later. DU was improved, and freedom from calculi was achieved by a facilitated URS procedure in all cases. The remaining two patients had stones in the lower calyx, and they opted for observation instead of URS. Of 18 patients for whom fragmentation alone could be performed, two required a second URS, and all 18 patients achieved stone-free status. On the other hand, stone-free status was achieved in 102 patients (81%) in the non-DU group who underwent primary URS. Ureteral mucosal injury was found in four cases (12.9%), and postoperative fever >38°C was observed in three cases (9.7%) in the DU group alone. No severe complications, such as septic shock or ureteral rupture, were recorded. All ureteral injuries were resolved upon second URS, and none of the patients in either group had hydronephrosis on CT imaging.

## 4. Discussion

Jones et al. first described that 11% of URS procedures with a 9.5/11 Fr semirigid ureteroscope had failed to reach the stones. For such cases, subsequent URS after stent indwelling was then facilitated [7]. Although smaller endoscopes could make failure to reach stones less frequent, even with a 6/7.5 Fr semirigid ureteroscope, 7.7–16.2% of URS procedures were still cancelled due to DU [6, 15, 16]. In this study, 31 of 156 (19.9%) cases were considered DUs. One possible reason for the higher rate of DU in this study is that we included patients in whom the ureteroscope managed to reach and fragment the stone following failed ureteral access sheath (UAS) insertion. If we excluded cases in which fragmentation alone could be performed, DU would number 14 cases (9.0%), which is comparable with previous studies. The reason why we applied a more inclusive definition was because more cases could inform the understanding of DU more comprehensively, in the belief that understanding factors contributing to unusual URS cases is important to reduce perioperative complications. Interestingly, the majority of DU cases in which only fragmentation could be performed achieved stone-free. We speculate that this is because of the passive dilatation effect of ureteral stents.

In this study, we demonstrated that a major axis <8 mm, negative traceability, smoking status, and absence of DM were independently predictive of DU, upon the multivariable analysis. In the DU group, the major axis was significantly smaller (8.8 mm vs. 10.9 mm). This is explained by the fact that patients with DU have some difficulty passing small stones that should spontaneously migrate through the normal ureter. In other words, small stones indicate the narrow lumen of the ureter. Fuller et al. also reported that patients with DU tend to have stones <10 mm [16]. One study investigated the relationship between CT urography and DU [15]. They reported that <50% ureteral opacification on CT urography was independently associated with an increased risk of access failure. Subjecting all patients to preoperative medical imaging by CT urography was difficult in terms of cost and the adverse effects of contrast media or radiation exposure. We alternatively verified the meaning of “traceability” in this study to resolve the disadvantages of CT urography. Traceability can be defined on plain CT imaging that all URS patients should undergo. Indeed, this new index is a remarkable predictor (OR: 7.565).

No previous studies have indicated that the absence of DM and current smoking are associated with DU. We focused on the medical history of DM, HT, DL, smoking, and cardiovascular or cerebrovascular disease and on the hypothesis that arteriosclerosis in the peripheral vessels of the ureteral wall might reduce compliance and cause DU. However, no other conditions except for current smoking was associated with DU. We speculate that this is because smoking more strongly induces arteriosclerosis than the other factors [17]. Interestingly, DM showed an effect that is contrary to our assumption. The absence of DM was also associated with DU. A possible reason for the lower risk of DU in patients with DM is increased urine flow through the ureter due to polyuria and passive dilatation of the ureteral wall due to neuropathy [18]. Although it was not significant in this study, younger age [16] was a potential factor. It might be speculated that the urethral wall becomes soft, and the connective tissue ultimately becomes loose with advancing age. Viers et al. reported that prior ipsilateral stone surgery [15] contributes to an 85% risk reduction of DU. We collected data on the history of urolithiasis on the affected side instead of just prior stone surgery for more inclusive analysis. Unfortunately, it was not a significant factor, based on our study (19.3% vs. 31.2%, *P*=0.193). Despite this, the non-DU group tended to have prior urolithiasis on the affected side. Stone location has also been reported to be associated with DU [16]. The structure of the rigid endoscope could explain this association. It usually has a thinner diameter in its leading edge; therefore, it is easier to reach the distal ureter than the proximal ureter. The present analysis was performed considering (1) renal stones alone and (2) ureteral stones alone/ureteral and renal stones in combination because we did not have enough cases with ureteral stones alone.

It should be noted that DU is just a relative notion of the ureteral diameter on the surgical approach, not a disease. An ultrathin, semirigid ureteroscope can reduce risk of DU [19], and a thinner endoscope may completely solve this problem for ureteral stones. However, to treat renal stones, a flexible ureteroscope is essential, and a UAS will still be useful. Indeed, a UAS will be applied in the era of robotic URS [20]. Therefore, predicting DU remains meaningful, even in the near future.

We also emphasize that diseases that can induce ureteral strictures must be differentiated from DU, such as tuberculosis, ureteral carcinoma, or ectopic endometriosis. Those diseases usually thicken the ureteral wall while difficulty in tracing a ureter is a finding of DU as demonstrated in this study.

Some limitations of our study should be mentioned. Foremost, care must be paid in drawing conclusions from our retrospective, single-center database with a relatively small number of cases. Second, questions about traceability should be addressed, namely, on objectivity and reproducibility of this attribute. To ensure objectivity and reproducibility, patients' information or the outcome of the URS procedure was not disclosed upon expert assessment. Furthermore, it should be noted that the point of negative traceability corresponds to a narrow point on the URS, which is not clear. Further studies are required to answer this question.

## 5. Conclusion

Patients with smaller stones, negative traceability, ongoing tobacco consumption, and the absence of diabetes mellitus were at higher risk of difficult ureters. In these patients, preoperative stenting may be considered.

## Figures and Tables

**Table 1:** Patients background.

		DU	Non-DU	*P*
*N*		31	125	
Age		55.1 (11.9)	59.3 (13.3)	0.107
Sex	Female	7 (22.5)	22 (17.6)	0.523
BMI (kg/m^2^)		24.3 (3.9)	24.8 (3.9)	0.581
Side	Left	15 (48.3)	68 (54.4)	0.548
Stone location	Renal alone	15 (48.3)	54 (44.0)	0.603
Major axis (mm)		8.8 (3.9)	10.9 (4.5)	0.018^*∗*^
Maximum Hounsfield unit		1202.5 (372.0)	1257.3 (355.3)	0.445
Hydronephrosis	Present	15 (48.3)	66 (52.8)	0.660
Traceability	Positive	15 (48.3)	104 (83.2)	<0.001^*∗*^
History	Urolithiasis of the affected side	6 (19.3)	39 (31.2)	0.193
	Abdominal surgery	3 (9.6)	22 (17.6)	0.282
	Smoking habit	16 (51.6)	54 (43.2)	0.399
	DM	2 (6.4)	29 (23.2)	0.105
	HT	12 (38.7)	68 (54.4)	0.118
	DL	8 (25.8)	29 (23.2)	0.760
	Cardiovascular/cerebrovascular disease	3 (9.6)	8 (6.4)	0.523

^*∗*^Statistical significance. BMI, body metabolic index; DL, dyslipidemia; DM, diabetes mellitus; DU, difficult ureter; HT, hypertension.

**Table 2:** Multivariate logistic regression models.

	Full				Reduced			
	OR	95% CI		*P*	OR	95% CI		*P*
		Lower	Upper			Lower	Upper	
Age	0.976	0.939	1.015	0.226				
Sex (female)	1.097	0.269	4.478	0.897				
BMI	1.037	0.889	1.209	0.648				
Major axis (>8 mm)	8.852	2.402	32.615	0.001^*∗*^	4.495	1.791	11.278	0.001^*∗*^
Stone location (renal alone)	3.945	0.997	15.604	0.050				
Maximum HU	1.001	0.999	1.003	0.365				
Hydronephrosis	2.259	0.636	8.025	0.208				
Urolithiasis of the affected side	0.539	0.157	1.846	0.325				
Abdominal surgery	0.642	0.135	3.045	0.577				
Traceability (negative)	7.947	2.324	27.171	0.001^*∗*^	7.565	2.693	21.248	<0.001^*∗*^
Smoking status	2.912	1.007	8.427	0.049^*∗*^	3.196	1.164	8.773	0.024^*∗*^
Absence of DM	8.110	1.167	56.374	0.034^*∗*^	5.813	1.121	30.142	0.036^*∗*^
HT	0.615	0.208	1.819	0.380				
DL	1.174	0.351	3.929	0.795				
Cardio/cerebrovascular disease	2.740	0.418	17.947	0.293				

^*∗*^Statistical significance. BMI, body metabolic index; CI, confidence interval; DL, dyslipidemia; DM, diabetes mellitus; HT, hypertension; OR, odds ratio.

## Data Availability

The datasets used during the current study are available from the corresponding author upon request.
